# Supplementary material for: The interactome of intact mitochondria by cross-linking mass spectrometry provides evidence for coexisting respiratory supercomplexes
Source: Mol Cell Proteomics. 2017 Dec 8;17(2):216–32. doi: 10.1074/mcp.RA117.000470 (PMC5795388; doi:10.1074/mcp.RA117.000470)
Supplement: Supplemental Data [file supp_RA117.000470_133922_0_supp_23978_fzffwf.zip › spectra_annotation/mito_spectra_annotation/92-1-8-1-3-1.pdf]

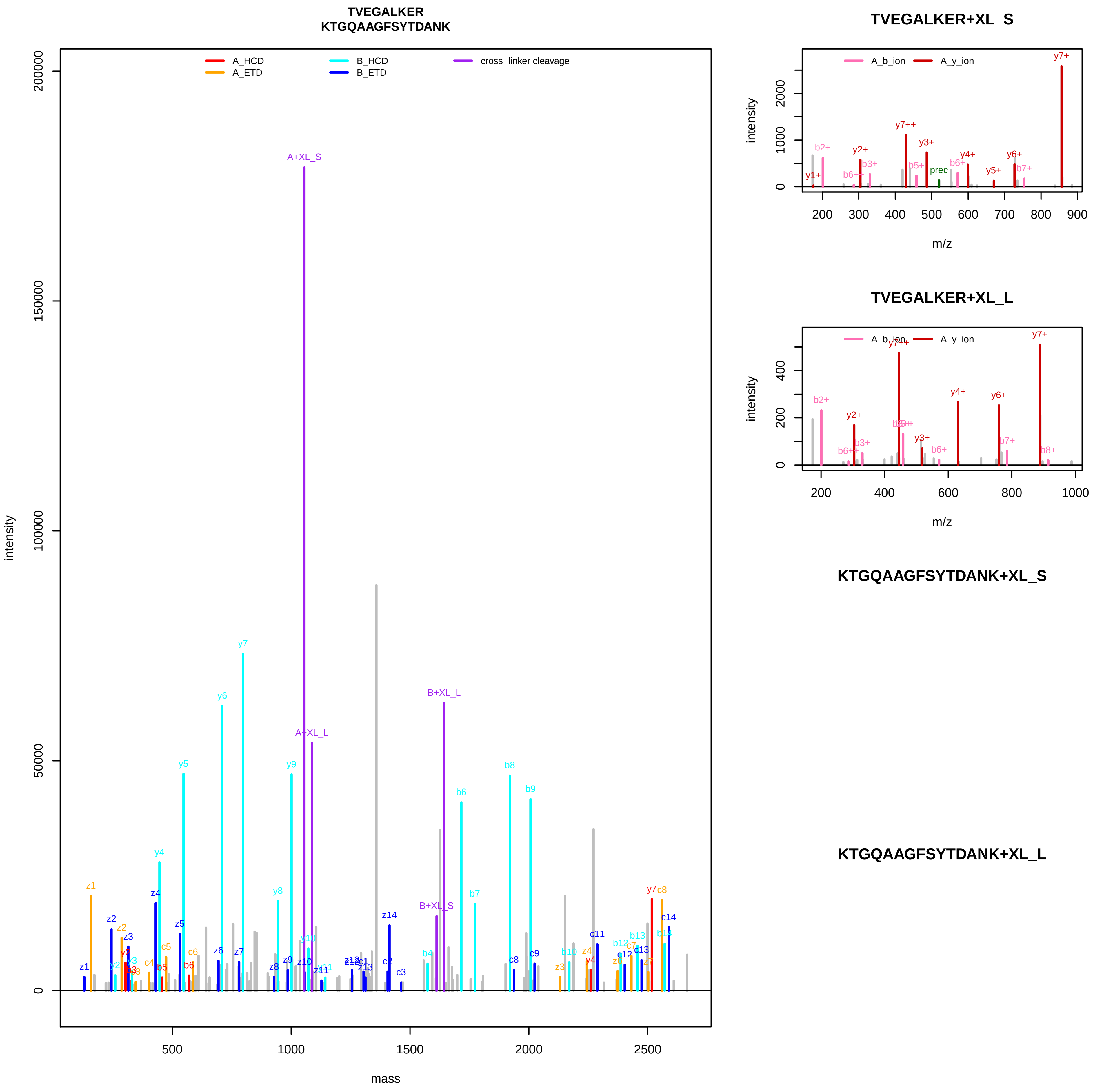
TVEGALKER
KTGQAAGFSYTDANK
A_HCD
A_ETD
B_HCD
B_ETD
cross−linker cleavage
A+XL_S
A+XL_L
B+XL_L
B+XL_S
intensity
mass
0
50000
100000
150000
200000
500
1000
1500
2000
2500
TVEGALKER+XL_S
A_b_ion
A_y_ion
y7+
y7++
y3+
y2+
b2+
b3+
b5+
b6+
prec
y4+
y6+
y5+
b7+
y1+
b6++
intensity
m/z
0
1000
2000
200
300
400
500
600
700
800
900
TVEGALKER+XL_L
A_b_ion
A_y_ion
y7+
y7++
b2+
y2+
y4+
y6+
y3+
b3+
b6+
b7+
b8+
b6++
intensity
m/z
0
200
400
200
400
600
800
1000
KTGQAAGFSYTDANK+XL_S
KTGQAAGFSYTDANK+XL_L
